# Impact of time pressure on software quality: A laboratory experiment on a game-theoretical model

**DOI:** 10.1371/journal.pone.0245599

**Published:** 2021-01-15

**Authors:** Dirk Basten, Marcel Müller, Marion Ott, Oleg Pankratz, Christoph Rosenkranz

**Affiliations:** 1 Cologne Institute for Information Systems, University of Cologne, Cologne, Germany; 2 School of Business and Economics, RWTH Aachen University, Aachen, Germany; Universidad Loyola Andalucia Cordoba, SPAIN

## Abstract

Research suggests the relationship between time pressure and software quality to be more complex than presumed. While software developers can adjust their output to improve observed performance at the expense of software quality, the latter has been found to increase with time pressure in case of work-pace dependent incentives. An untested, but widely disseminated game-theoretical model seeks to resolve this contradiction and hypothesizes that high rates of time pressure avoid so-called ‘shortcuts’, which occur in the form of imperfections induced by developers to meet unrealistically tight deadlines. We conduct two laboratory experiments to empirically test this model for the first time. Our results corroborate the model with regard to its suggestion that shortcuts can be reduced if developers perceive unrealistic deadlines as ever-present. However, we also show that the actual critical probability of unrealistic deadlines–the point at which shortcut taking is drastically reduced–is above the theoretical one. Although final conclusions on the impact of time pressure on software quality remain to be drawn, our results suggest that–considering the contingencies of our study–time pressure helps in striving for quality in software projects.

## Introduction

*Time pressure* that results from too optimistic project schedules likely impairs software quality in software development [1], and time pressure even is deemed “the single greatest enemy of software engineering” [2]. However, the findings of empirical research [e.g., 3–5] on the impact of time pressure (i.e., the perceived tightness of deadlines) are inconclusive. For example, studies find both a negative and a positive impact of time pressure on work performance [6]. Additionally, research has identified an inverted U-shape relationship between time pressure and performance [6, 7], proposing this relation to depend on the level of time pressure.

In the software project management domain, research on time pressure has shown that developers under time pressure do not necessarily work better, they just work faster [8], and that time pressure can reduce software quality [4]. On the other hand, decision quality and speed under restrictive deadlines have been found to increase when work-pace dependent incentives are given (e.g., higher payoff if work is faster) [3]. These different findings highlight that many aspects of time pressure’s impact on the software development process remain unexplained [9]. For example, these studies do not consider developers’ option to report their inability to meet a deadline and to ask for an extension of time.

A game theoretic model first proposed by Austin [10] explores the relationship between time pressure and software quality when missing a deadline or reducing quality is a decision taken by the developer. Time pressure is modelled as the probability with which deadlines of assigned tasks turn out to be unrealistic. (In our study, we follow Austin’s approach of modeling time pressure as the probability with which deadlines of assigned tasks turn out to be unrealistic rather than as the tightness of a specific deadline.) The model identifies a deadline-setting strategy of *increasing* time pressure to enhance software quality. In this model, so-called ‘shortcuts’ are defined as software imperfections that developers deliberately induce to save time and meet a tight deadline. As a consequence of taking such shortcuts, software developers do not have to report delays to their supervising project managers (who are presumed to not be able to detect such shortcuts). While it stands to reason that reduced time pressure can decrease the number of shortcuts, Austin’s counterintuitive hypothesis is that high time pressure (such that the probability of unrealistic deadlines is sufficiently high) may be better. According to the model, continuously high levels of time pressure result in a state in which the shortage of time is so ever-present that reporting delays is destigmatized, ultimately leading to less shortcut taking and thus higher software quality.

Despite its widespread dissemination and discussion [7, 11–18], the model has not been empirically tested so far. To close this gap, we design, conduct, and evaluate two laboratory experiments to test the model. We show that a higher probability of time pressure indeed leads to less shortcut tasking, and presumably higher software quality. In doing so, we contribute to software project management literature in two important ways. First, we provide empirical support for Austin’s model and empirically corroborate for the first time that high levels of time pressure indeed are a mechanism that helps abandon the stigmatization of reporting delays. Second, we contribute to theory conceptualizing the complex relationship between time pressure and software quality in more detail. Our findings advance our understanding of Austin’s model by suggesting that the actual critical probability of unrealistic deadlines–the point at which shortcut taking is drastically reduced–is above the theoretical one. Both insights have a high practical relevance to software project management by questioning common approaches to deadline setting.

The remainder of the paper is structured as follows. Next, we describe related work concerning time pressure in organizational contexts and introduce Austin’s model. We then explain the design of our two experiments and present the respective results. Subsequently, we discuss implications of our study as well as directions for future research. Our paper ends with a short conclusion.

## Related work and theoretical background

### Time pressure in organizational work contexts and software development

*Time pressure*–defined as the perception that the time available to complete a task is scarce in relation to the demands of the task [19, 20]–is common in organizational settings [21–23]. However, research is inconclusive concerning the relation between time pressure and employees’ behavior and performance [6]. Diverse results comprise indicators that time pressure and work performance have a positive [e.g., 20, 23, e.g., 24], negative [e.g., 25, 26], or an inverted U-shaped relationship [6]. These works also suggest that the level of time pressure is decisive for the type of impact: whereas moderate time pressure is necessary to ensure motivation, high or no time pressure leads to distraction or lack of stimulation, respectively.

The general inconclusive findings concerning time pressure hold for the domain of software development as well, finding no effect [27], positive effects [4], or an inverted U-shape effect [7].

On a more detailed conceptual level, it is traditionally presumed that tight deadlines lead to time pressure [8], which in turn tempts software developers to take *shortcuts* (i.e., reductions of software quality) since reporting delays is typically stigmatized in software projects [10]. People under time pressure do not necessarily work more efficiently (i.e., get more done in a smaller amount of time), they simply reduce the amount of work that needs to be done, which leads to worse software quality [3, 10], especially if performance cannot be observed or verified [28]. This typically applies to settings in which a software developer (an agent) can manipulate outputs at the expense of project quality without the supervising project manager (a principal) noticing this manipulation. Thus, agents can hazard the consequences of reducing software quality by taking quality-compromising shortcuts without having to fear punishment from their principal.

The usual approach to reduce shortcut taking is to add slack to the project schedule [10]. Such safety buffers are intended to reduce risks of wrong estimates [29, 30]. However, they lead to inefficiencies and also bear the risk that developers, knowing of the actual real estimates, misapply given buffers to reduce working speed [31], or that they engage into ‘gold plating’ [32], meaning that developers design, implement, test, document, and support ‘fancy’ or new features that are not required and lengthen the schedule.

### Austin’s agency model

A game-theoretical model that is counter-intuitive at first glance, because it actually turns the usual slack-based approach on its head and claims a positive effect of higher levels of time pressure on software quality, has been proposed by Austin [10]. The model conceptualizes the “software quality under time pressure” scenario as follows. Two *agents* (i.e., software developers) compete for the favor of a *principal* (i.e., the project manager). This includes rewards (e.g., promotions, pay raises, future business) from the principal to whom they report at regular intervals. The players in the game are these two agents, who independently decide on whether to report their inability to meet a given deadline or to take a shortcut to meet it. The prevailing deadline-setting policy is represented by the *probability p > 0 of a software developer being confronted with an unrealistic deadline* and is taken as given. The central conclusion of the model is that a critical value of p (henceforth referred to as *p*_*crit*_) exists; for p < p_crit_, the number of shortcuts taken increases with p, whereas for p ≥ p_crit_, shortcuts are completely avoided.

According to Austin [10], shortcuts “are decisions made in private [by software developers] that are motivated by a desire to stay on schedule, but are not in the best interests of the project”. Shortcuts can lead to serious software failure during runtime, and software developers are usually not (fully) aware of the possible consequences when they decide to take such shortcuts. It is also important to mention that software developers with concerns for quality who take a shortcut under time pressure would not do so in case of alleviated pressure. In addition, it is presumed that software developers do not have to fear personal consequences when taking shortcuts since it is difficult for non-specialists (such as the principal) “to trace complex system problems to causal sources” [10].

The project manager assigns software projects to the two developers. The respective developer is aware of her own deadline situation but does not know about the deadline situation of the other agent. (We refer to managers and software developers as ‘she’ or ‘her’, representing both sexes.) The deadline situations of the two agents are independent but are subject to the same probability p. In case of a realistic deadline, the respective agent delivers high quality with no decision to make. Otherwise, she must decide between delivering high quality (H), which results in missing the deadline, and taking a shortcut, which results in low quality (L) while meeting the deadline. The following penalties define the payoff of each outcome:

1. Concern for career (penalty C): Occurs in the case of one agent ‘looking bad’ in the eyes of the principal because she is behind schedule while the other agent is not. The penalty arises for an agent who chooses H in an unrealistic deadline situation, while the other agent either has an unrealistic deadline and takes a shortcut (i.e., chooses L) or has a realistic deadline.
2. Concern for quality (penalties Q1 and Q2): Represent the negative effect of poor software quality on the company’s image, which is presumed to reflect equally on all software developers in this company. The penalty arises for both agents if one (penalty Q_1_) or both (penalty Q_2_) have an unrealistic deadline and take a shortcut (L).

**Fig 1:**
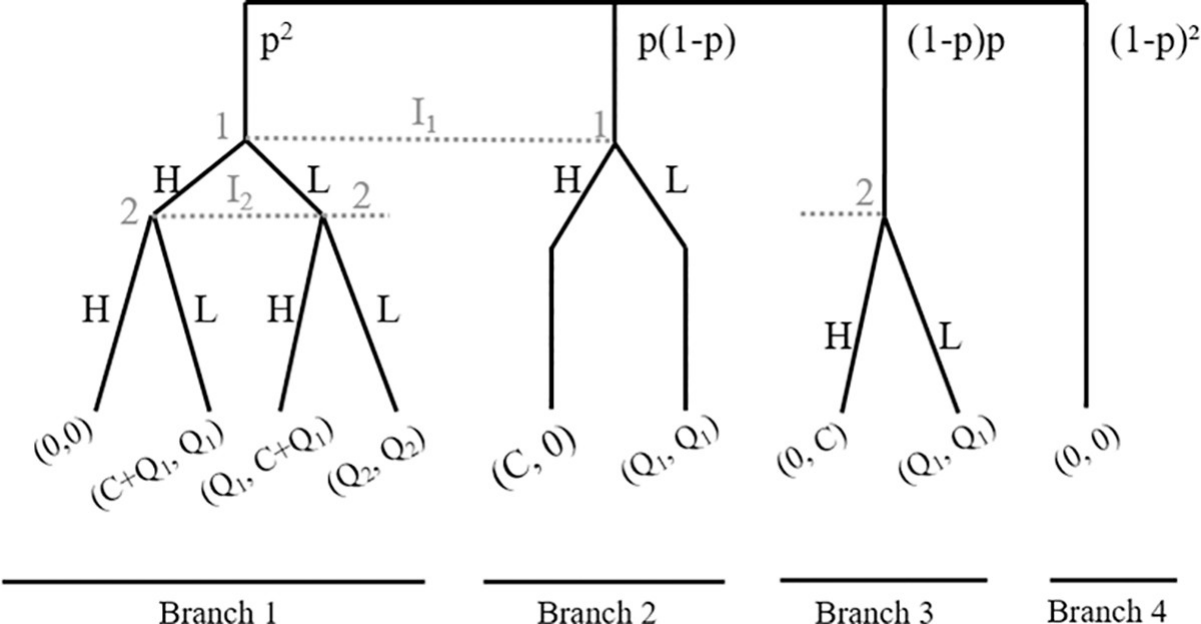
Extensive-form representation of Austin’s game. Austin’s hypothesis on software quality is as follows. Agents will play according to the unique NEQ (L, L) for p < p_crit_. For p ≥ p_crit_, agents will play the payoff-dominant NEQ (H, H). Thus, conditional on having an unrealistic deadline, for low p all decisions will be L and for high p all decisions will be H, that is, the percentage of H-decisions increases in p.

In the following, we presume that C > Q_2_ > Q_1_ > 0. Fig 1 displays the extensive form of Austin’s game. The agents’ payoffs (u_1_, u_2_) are shown on the respective leaves of the game tree. Note that the information sets I_1_ and I_2_ represent the missing information of the respective agent about the deadline situation of the other. Four situations exist:

1. Both agents face an unrealistic deadline (branch 1, with probability p^2^): In this case, both agents must choose simultaneously between high quality (H) and taking shortcuts (L).
2. Only agent 1 faces an unrealistic deadline (branch 2, with probability p(1-p)): In this case, only agent 1 must choose between H and L.
3. Only agent 2 faces an unrealistic deadline (branch 3, with probability (1-p)p): Analogous to situation 2.
4. No agent faces an unrealistic deadline (branch 4, with probability (1-p)^2^).

However, due to agents’ lack of information, their single decision for their information set (i.e., if their deadline is tight) cannot depend on the other agent’s deadline situation.

To deepen the understanding of the model in view of interpreting experimental data, in the supporting information S1 Appendix we extend Austin’s analysis (1) by classifying the game depending on its parameters into three types, (2) by exploring risk-averse agents, and (3) by analyzing repeated play of his one-shot game.

This predicts the following rate of shortcut taking (r). Define r as the number of shortcuts taken divided by the total number of deadline situations, and assume the game is played k times by n pairs of agents. The rate r is zero if there are no deadlines and increases equally with the probability of an unrealistic deadline p for p ≤ p_crit_, because every agent will seize any unrealistic deadline she faces (play L), such that r = 2npk/(2nk) = p. At p = p_crit_, the rate abruptly drops to zero because every agent solely plays H for p ≥ p_crit_. Thus, r and p have a non-monotonic relationship.

Austin’s prediction has not been empirically tested so far. Therefore, we test for the first time in two experiments Austin’s hypothesis that agents play (L, L) in case this is the unique NEQ and play (H, H) when it is the payoff-dominant NEQ.

## Experimental studies

### General setup

Our research includes two experiments. The first is designed to analyze the effects on time pressure on software quality in an abstract setting (i.e., the software development setting was described to participants, who then decided between abstract options facing purely financial tradeoffs; see *Study 1*). This is the common approach in laboratory experiments on behavior in economics and psychological studies. The second experiment focuses on the actual behavior and includes real programming tasks that the participants were requested to solve under conditions of (un-)realistic deadlines (see *Study 2*).

The goal of the experiments is to empirically test Austin’s predictions and thus to test the following hypothesis: “*For p>0*, *higher probabilities of unrealistic deadlines reduce shortcut taking in software projects when exceeding a critical threshold compared to lower probabilities*.*”* Our experiments are based on the relation between the probability p of being assigned a task with an unrealistic deadline (independent variable) and either the decision (Study 1) or the action (Study 2) taken by a software developer (dependent variable)–either taking a shortcut (L) or reporting a delay (H) if facing an unrealistic deadline. Both variables are categorical. While the dependent variable can be either L or H, p as the probability of an unrealistic deadline, for which the impact on the decision of strategy (H or L) is to be investigated, depends on the parameters Q_1_, Q_2_, and C.

In designing and planning our experiments, we followed the guidelines of Wohlin, Runeson [33]. The experimental sessions lasted about 90 minutes. Participants for both experiments were recruited among students of an undergraduate course on software development at a German university. All students were close to graduation and finishing the course. During the software development course, the students had to develop a software product jointly with customers from industry. This experience of the students ensured a high-quality pool of participants. This is also evidenced by the self-reported skills in programming (mean 3.18, st.d. 0.81 on a five-point Likert scale) and HTML development (mean 2.57, st.d. 1.25). Although our participants have both project management and programming experience gained in the software development course, we acknowledge that they do not have the longstanding management, programming, and testing experience of professionals in industry. However, in software development, students have been shown to be suitable as representatives for professionals and that their results are generalizable [34]. This is especially the case if students are last-year software engineering students and if performed tasks are in the context of maturity and understanding of dependencies and relationships in software development [35]. Our setting fulfills both conditions. Moreover, students are easy to recruit and can be remunerated with a smaller amount compared to professionals [34, 35]. Each subject participated in only one treatment and in only one of the experiments (between-subject design, random assignment). For participating in our experiments, the students were asked to sign the written informed consent that is used for all experiments conducted in the laboratory at the corresponding author’s university. The consent defines the ground rules such as being paid as well as guaranteeing the anonymity of the participants and data privacy issues. All participants gave written consent.

Austin implies a scenario in which two agents interact with each other for several rounds, where one round represents one deadline situation. The payoff of subjects is important in order to incentivize proper decision-making. Each subject was awarded eight points per round, which represented the earnings in the given round. The realized penalties 0, Q_1_, Q_2_, C, or C+Q_1_ were then subtracted accordingly. The maximum penalty to be obtained in a single round was C+Q_1_ = 6; therefore, eight points were chosen as the earning amount in order to assure positive payoffs. Payoff points were converted into Euro after the sessions to determine the final earnings of the participants.

In both experiments, we established four treatments (p, Q_1_, Q_2_, C), which only varied in p. In a test session (with students working at the authors’ department and not being involved in the actual experiment), with parameters (Q_1_, Q_2_, C) = (1, 2, 3) implying p_crit_ = 2/3 and two treatments with p = 0.4 and 0.9 both did not lead to coordination to H. This observation is in line with the analysis (see S1 Appendix), showing that risk aversion might increase the actual value of p_crit_. Therefore, (Q_1_, Q_2_, C) = (2, 3, 4) were chosen in order to realize a lower p_crit_ = 0.5. The selected values of Q_1_ = 2, Q_2_ = 3, and C = 4 define p_ps_ = 0. As a result, for p < p_crit_ the game can be categorized as a Prisoner’s Dilemma and for p ≥ p_crit_ as a Stag Hunt coordination game (see S1 Appendix for the types of games and, e.g., Andreoni and Miller [36], Cooper, DeJong [37], and dal Bo [38] for experiments on the Prisoner’s Dilemma, and Devetag and Ortmann [39] for an overview of experiments on the Stag Hunt).

For our subsequent data analyses, we used IBM SPSS Statistics 24 and R version 3.4.2 with the package “mlogit”.

### Study 1

#### Design

The subjects were requested to make strategic decisions concerning whether they would choose high or low quality for given (un)realistic deadline situations. The experiment’s setting, instructions, procedure, and control questions are given in supporting information S2 Appendix. The study was conducted using the z-Tree software package [Zurich Toolbox for Readymade Economic Experiments; cf. 40]. We selected p = 0.4, 0.6, 0.75, and 0.9, which predominantly lay above p_crit_ = 0.5. While p = 0.4 and 0.6 are chosen to lie evenly and closely around p_crit_, p = 0.75 and p = 0.9 are chosen to make coordination to H more attractive if p = 0.6 (which should yield H, as predicted by Austin) is not high enough (based on insights from an early test session).

We sought 12 participants per treatment (i.e., 48 participants in total), of which nine were female and 39 were male. We considered this sample adequate since 44 participants would be sufficient for a power level of 0.8. Since our study relies on the relation between two categorical variables, this consideration is based on a goodness-of-fit test for the Pearson chi-square tests, calculated with the software tool G*Power (alpha = 0.05; degrees of freedom = 3; effect size = 0.5). The effect size is a modest estimate compared to effect sizes in software engineering experiments in general [41] and other software testing experiments in particular [1].

Subjects were randomly assigned to treatment groups by drawing a number from an urn and then randomly matched to groups of two subjects within the treatment for the duration of 24 rounds (i.e., a partner design in a repeated game is set up). Afterwards, new groups were randomly generated, assuring a different workmate for each participant, and another 24 rounds were implemented. The number of 24 rounds surfaced from the pretest and was chosen as a compromise between allowing the low-p treatment group (in which the occurrence probability of an unrealistic deadline is only 40%) enough rounds for coordination and preventing the high-p treatment groups from getting bored, which might have resulted in inattentive decisions. After each round, participants learned whether their other group member provided low or high quality but did not learn whether that group member had a realistic or an unrealistic deadline.

Participants were paid a show-up fee of 2.50 Euro plus the amount earned according to their decisions in the experiment. In order to determine the conversion factor from points to Euro, the expected numbers of points were roughly estimated and a conversion factor of 1/20 Euro/points was chosen in order to yield an average payoff of 10 Euro per hour. Based on the payoffs given in Table 1, consistent play of (L, L) [(H, H)] leads to a player’s total expected points of (314, 286, 267, 251) [(338, 338, 348, 367)] for p = 0.4, 0.6, 0.75, and 0.9. (e.g., if both players in the 0.6 treatment choose L in each round, the expected payoff is calculated as follows: number of rounds*(constant earning—expected penalty) = 48*(8-(p^2^Q_2_+2p(1-p)Q_1_)) = 48*(8-(0.6^2^*3+2*0.6*(1–0.6)*2)) = 286.08). Assuming L is unlikely to be chosen for high p, and further assuming that choosing H should result in higher penalties through imperfect coordination, a constant conversion factor was presumed to yield roughly the same payoff for all treatment groups. The participants’ final payment was between 14.60 Euro and 20.80 Euro with a mean of 18.26 Euro (median 18.20 Euro).

**Table 1:** Normal form of Austin’s game.

	H	L	
H	p(1-p)C	pQ_1_	
	p(1-p)C	p(C+Q_1_)	
L	p(C+Q_1_)	p^2^Q_2_+2p(1-p)Q_1_	
	pQ_1_	p^2^Q_2_+2p(1-p)Q_1_	
Agent 1: Row player Agent 2: Column player		Payoffs in cells:	u_2_ u_1_

#### Descriptive statistics

**Fig 2:**
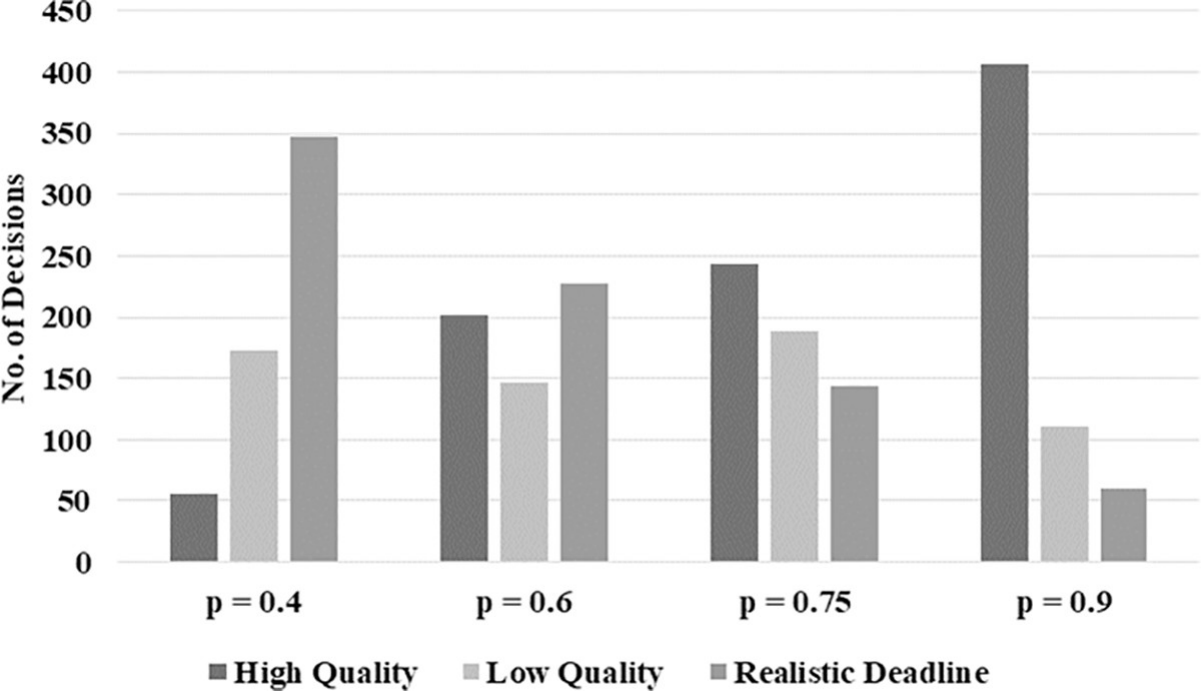
Distribution of unrealistic deadline (high-quality and low-quality decisions) and realistic deadline situations per treatment (n = 576).

Fig 2 shows the distribution of realistic and unrealistic deadline situations for the four treatments with varying values of p (total number of deadline situations per treatment n = 576). For unrealistic deadline situations, we further differentiate between the decisions made by the subjects concerning high (H) or low (L) software quality. The decreasing number of realistic deadline situations (from left to right) is a direct consequence of the increasing probability p of software developers being assigned an unrealistic deadline (from p = 0.4 to p = 0.9). As can be seen along the increasing probability of being confronted with an unrealistic deadline, the number of decisions for H increases while the one for L decreases. However, there is one exception: the transition from p = 0.6 to p = 0.75 (we discuss this non-linear relationship in the following section).

**Fig 3:**
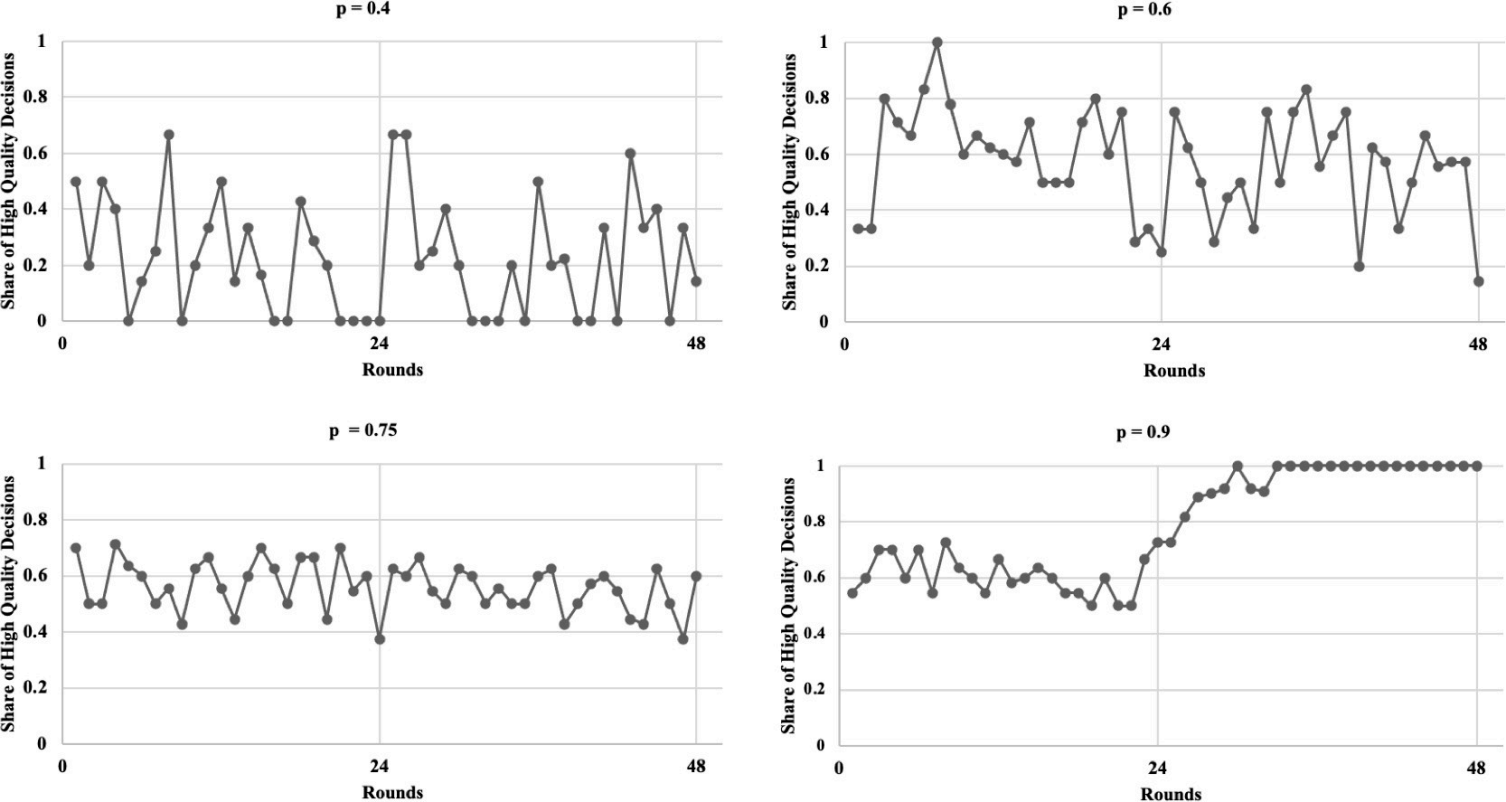
Share of high-quality decisions (unrealistic deadlines only) depending on the probability of facing an unrealistic deadline.

**Fig 4:**
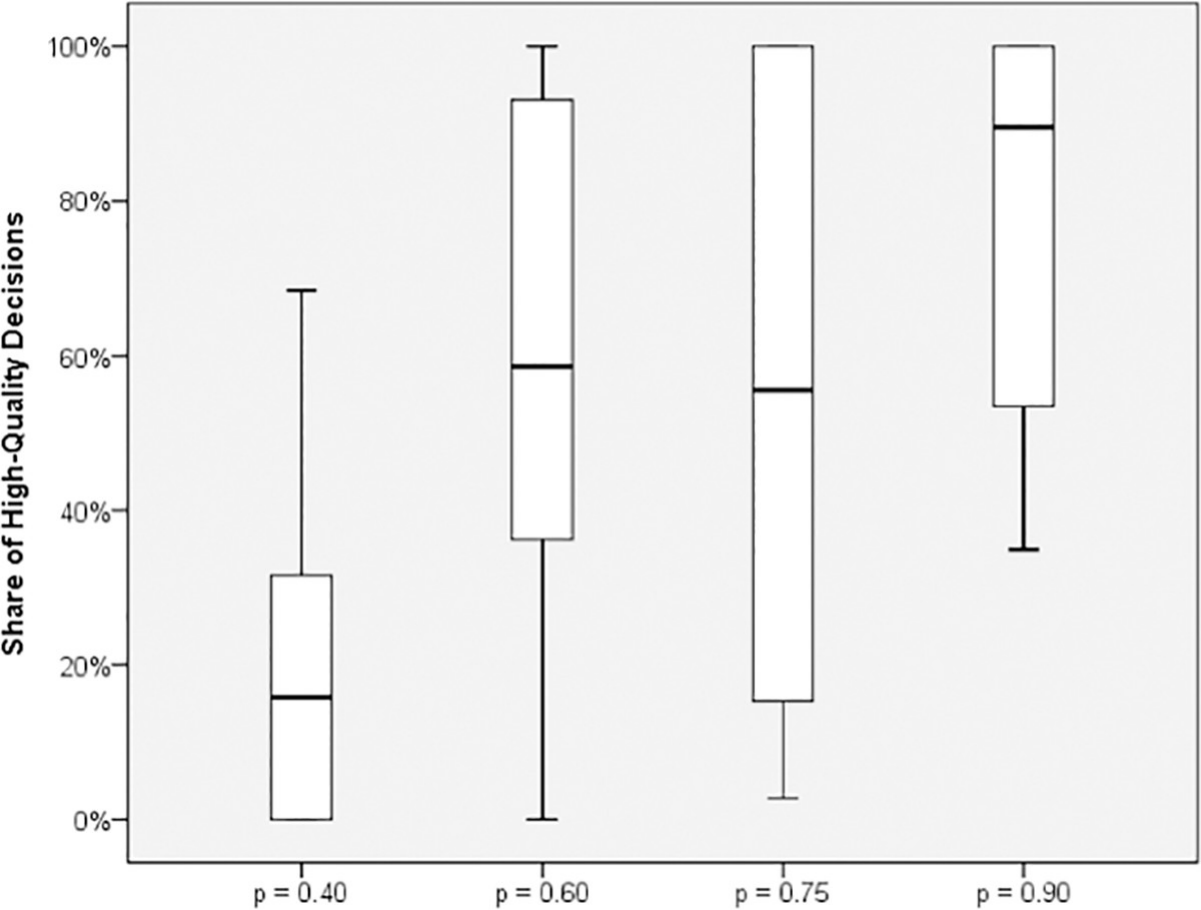
High-quality rate plotted for the four treatments.

**Fig 5:**
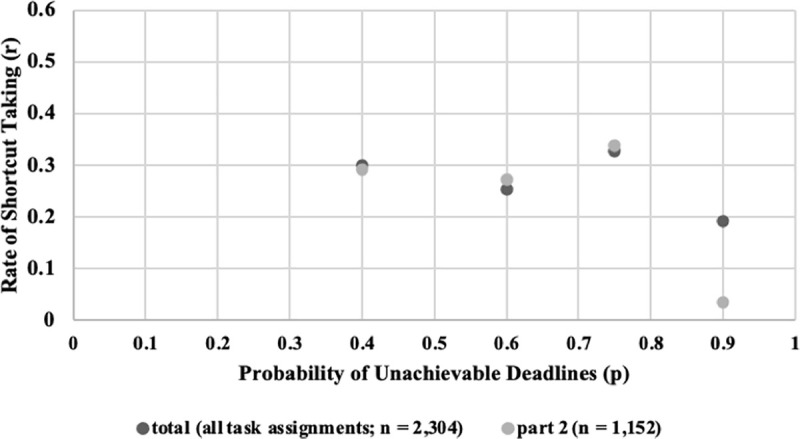
Relation between probability of unrealistic deadlines (p) and rate of shortcut taking (r).

Fig 3 illustrates the share of H decisions (number of H decisions divided by the number of unrealistic deadlines) per round for each treatment. A clear trend of an increasing share of H decisions along with p is found. For p = 0.4, the decisions are mostly L. In contrast, we observe predominantly H decisions for p = 0.9. In particular, the observation of both subjects playing H is at about 60% for rounds 1–24 (henceforth denoted as part 1) but increases to almost 100% in rounds 25–48 (henceforth denoted as part 2), that is, after regrouping. Strong fluctuations are observed especially for p = 0.4 and 0.6, resulting from averaging over subjects. Fig 4 shows the respective box plots. A clear trend of increasing H decisions along with p is visible according to the median values and variances. Surprisingly, from p = 0.6 to p = 0.75, the median values concerning the share of high-quality decisions remain nearly constant. Only at p = 0.9, H is the predominant decision. Finally, Fig 5 shows the relation between the probability of unrealistic deadlines (p) and the rate of shortcut taking (r, i.e., number of shortcuts taken divided by the total number of deadline situations). We show the relation for both the total experiment and part 2 (i.e., the participants already being familiar with the game).

#### Tests of significance

For the analysis, we considered the cases in which participants faced unrealistic deadlines, that is, the cases in which the participants needed to decide between high (H) and low quality (L). Since our data includes two categorical variables, we conducted a Pearson chi-square test [42] to test whether the differences between the treatments of varying probabilities of unrealistic deadlines are significant [43]. Since each subject participated in one of the treatments only, we can ensure the independence of residuals. With the lowest expected frequency count amounting to 92.3 (see Table 2), our data fulfills the criterion of expected frequencies.

**Table 2:** Contingency table for chi-square test (SPSS output).

			Quality		
			H	L	Total
Treatment	p = 0.4	Count	56	172	228
		Expected Count	135.7	92.3	228
		% within Treatment	24.6%	75.4%	100%
		% within Quality	6.2%	27.9%	15%
		% of Total	3.7%	11.3%	15%
		Std. Residual	-6.8	8.3	
	p = 0.6	Count	202	146	348
		Expected Count	207.1	140.9	348
		% within Treatment	58.%	42.0%	100%
		% within Quality	22.3%	23.7%	22.8%
		% of Total	13.3%	9.6%	22.8%
		Std. Residual	-0.4	0.4	
	p = 0.75	Count	243	189	432
		Expected Count	257.1	174.9	432
		% within Treatment	56.3%	43.8%	100%
		% within Quality	26.8%	30.6%	28.3%
		% of Total	15.9%	12.4%	28.3%
		Std. Residual	-0.9	1.1	
	p = 0.9	Count	406	110	516
		Expected Count	307.1	208.9	516
		% within Treatment	78.7%	21.3%	100%
		% within Quality	44.8%	17.8%	33.9%
		% of Total	26.6%	7.2%	33.9%
		Std. Residual	5.6	-6.8	
Total		Count	907	617	1524
		Expected Count	907	617	1524
		% within Treatment	59.5%	40.5%	100%
		% within Quality	100%	100%	100%
		% of Total	59.5%	40.5%	100%

There is a significant association [Fisher’s exact test; 44] between the probability of software developers being confronted with an unrealistic deadline and the rate of shortcut taking (χ^2^ (3) = 202.192, p < 0.000, Cramer’s V = 0.36). Based on the odds ratio (i.e., effect size), the odds of choosing H were 11.34 times higher in case of p = 0.9 compared to p = 0.4. All odds ratios are shown in Table 3. The general trend is: the higher the probability of being confronted with an unrealistic deadline the more likely do subjects decide to deliver high quality (in cases they are actually confronted with an unrealistic deadline).

**Table 3:** Odds ratios among the four different treatments.

	p = 0.4	p = 0.6	p = 0.75
p = 0.6	4.25	-	-
p = 0.75	3.95	0.93	-
p = 0.9	11.34	2.67	2.87

To confirm our findings, we further analyzed the data by considering the share of high-quality decisions of her high/low quality decisions (i.e., we interpreted our categorical variable as the percentage of each participant choosing high quality). Based on the data for each group of two participants in parts 1 and 2 of our experiment, we used the Jonckheere-Terpstra test [45, 46] to assess whether a trend in the data is apparent. Since we presume that effects needed some rounds to level off, we used the data from rounds 13–24 (part 1) and 37–48 (part 2) for this assessment. The result shows a trend with significance for part 1 (p < 0.10, n = 24, df = 3) and part 2 (p < 0.01, n = 24, df = 3). (When considering all rounds, the p-values amount to 0.105 for part 1 and 0.023 for part 2.) This indicates that the high-quality decisions as well as the high-quality collaboration in teams increase along with p.

### Study 2

#### Design

The subjects were requested to solve simple HTML (Hypertext Markup Language) programming tasks. All participants at least were familiar with HTML and had used it in the past (see Section “General Setup”). The experiment’s setting, instructions, procedure, control questions, tutorial, and tasks are given in supporting information S3 Appendix. The study was conducted using the oTree software [47]. Based on our insights from the first study, we selected p = 0.4, 0.5, 0.7, and 0.9. While we kept p = 0.4, we tested the theoretical value for p_crit_ (i.e., 0.5) as well as p = 0.7 and p = 0.9 in the second study.

We sought 32 participants per treatment (i.e., 124 participants in total as four participants did not show up for the treatment with p = 0.5), of which 23 were female, 100 were male, and one participant preferred not to answer the respective question. Since the same power analysis applies and this sample size is above the one from the first study, the sample size is adequate.

Subjects were randomly matched to groups of two subjects for the duration of 11 rounds (i.e., a partner design in a repeated game is set up). The number of 11 rounds was chosen as a compromise along the lines of Study 1. The number of rounds is lower than in Study 1 and there is no second series because the real effort task in Study 2 requires additional time and attention, and places cognitive strains on the participants that are absent from Study 1. In the first 10 rounds, each participant was confronted with realistic and unrealistic deadlines according to the treatment to get used to the scenario (see below). The participants were told that the experiment would include 10–15 rounds, and the end was announced after the last round only. Designing the experiment this way was intended to avoid potentially deviating behavior in the last round: several participants in Study 1 acted differently in their last round and provided us with the reason in the post-experimental feedback. Reportedly, they acted on purpose since the competitor would not have the chance for “taking revenge”. We thus aimed to rule out this effect by keeping the participants unaware of what their last round would be. After each round, participants learned whether their other group member provided low or high quality but did not learn whether that group member had a realistic or an unrealistic deadline.

Participants were paid a show-up fee of 4.00 Euro (due to a change of conditions of the laboratory used) and a conversion factor of 1/6 Euro/points was chosen in order to yield an average payoff of 10 Euro per hour. Consistent play of (L, L) [(H, H)] leads to a player’s total expected points of (72, 69, 63, 57) [(77, 77, 79, 84)] for p = 0.4, 0.5, 0.7, and 0.9. Again, a constant conversion factor was presumed to yield roughly the same payoff for all treatment groups. The participants’ final payment was between 10.70 Euro and 18.00 Euro with a mean of 15.76 Euro (median 16.00 Euro).

Since the programming tasks required the participants to develop a website in HTML, we provided a respective tutorial (see S3 Appendix).

#### Descriptive statistics

**Fig 6:**
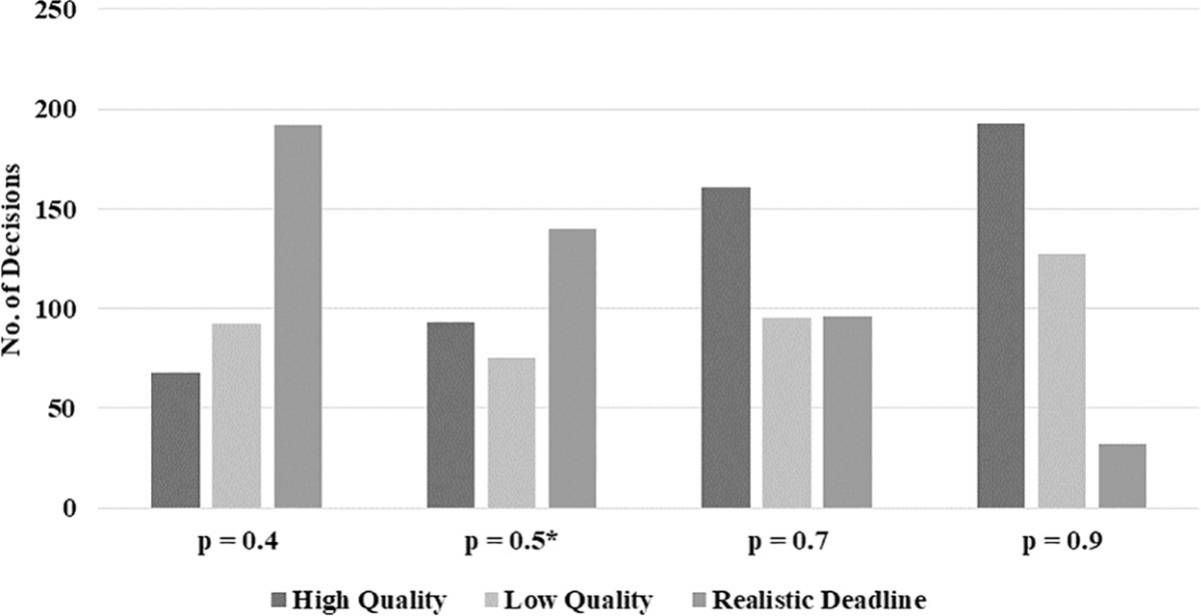
Distribution of unrealistic deadline (high-quality and low-quality decisions) and realistic deadline situations per treatment (n = 352, *n = 308).

Fig 6 shows the distribution of realistic and unrealistic deadline situations for the four treatments with varying values of p. For unrealistic deadline situations, we further differentiate between the decisions made by the subjects concerning high (H) or low (L) software quality. The decreasing number of realistic deadline situations (from left to right) is a direct consequence of the increasing probability p of software developers being assigned an unrealistic deadline (from p = 0.4 to p = 0.9). Along the increasing probability of being confronted with an unrealistic deadline, the number of decisions for H increases while the one for L does not decrease.

**Fig 7:**
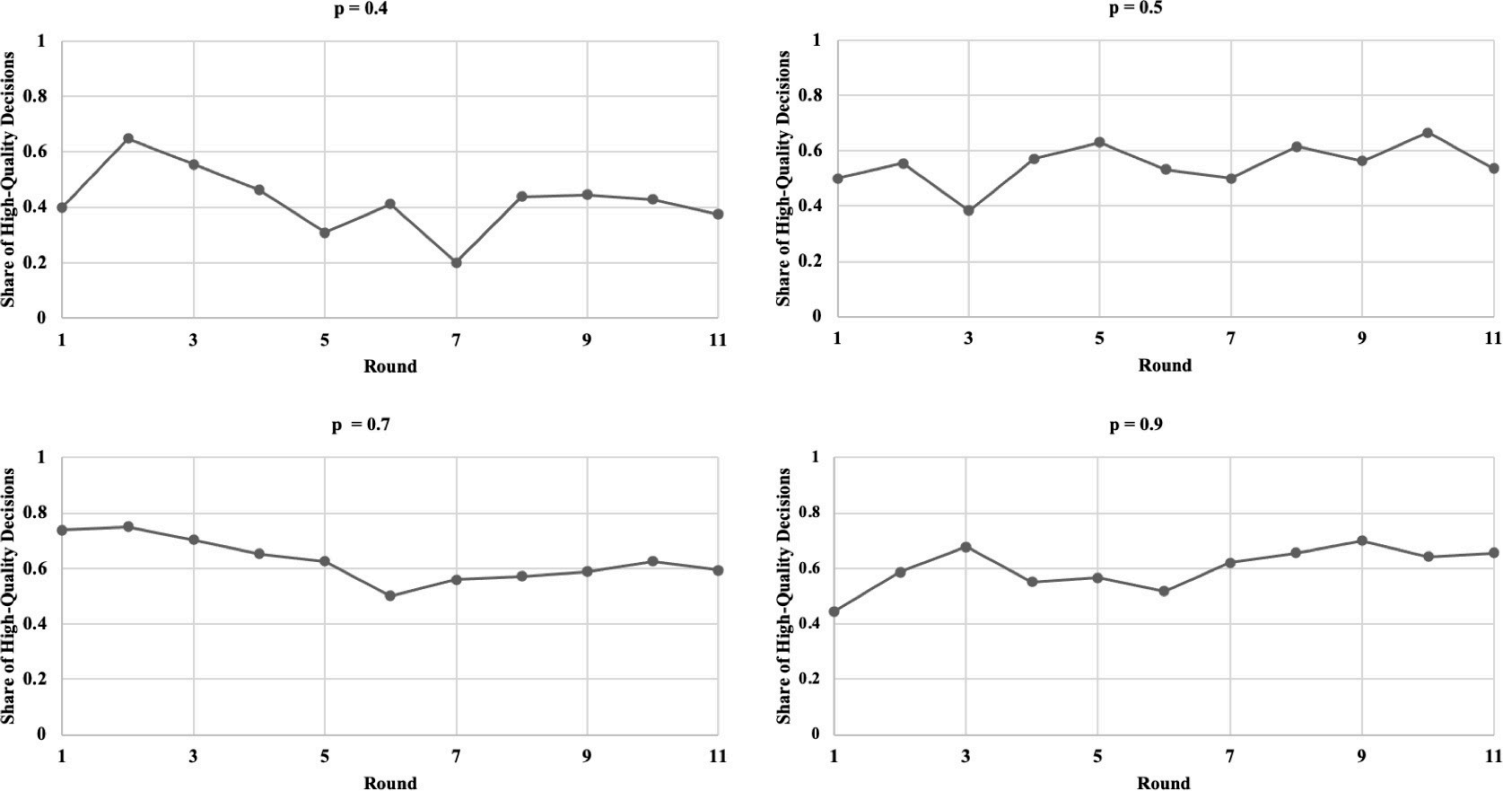
Share of high-quality decisions (unrealistic deadlines only) depending on the probability of facing an unrealistic deadline.

**Fig 8:**
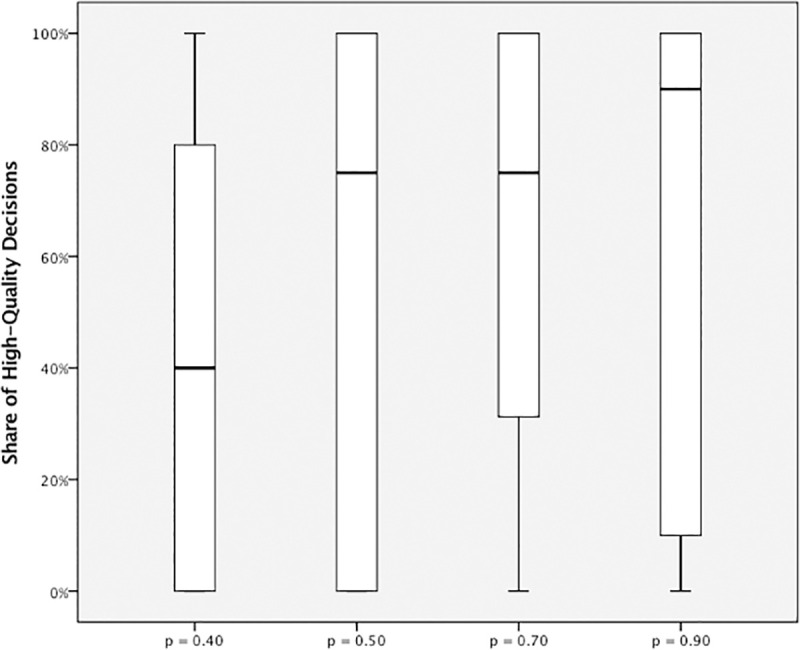
High-quality rate plotted for the four treatments.

**Fig 9:**
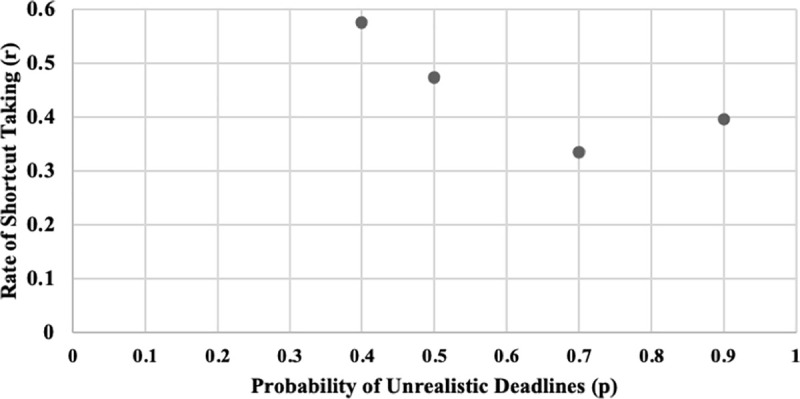
Relation between probability of unrealistic deadlines (p) and rate of shortcut taking (r).

Fig 7 illustrates the share of H decisions (number of H decisions divided by the number of unrealistic deadlines) per round for each treatment. Fluctuations are observed for all four treatments. Fig 8 shows the respective box plots. A trend of increasing H decisions along with p is visible according to the median values. Surprisingly, from p = 0.5 to p = 0.7, the median values concerning the share of high-quality decisions remain constant. Finally, Fig 9 shows the relation between the probability of unrealistic deadlines (p) and the rate of shortcut taking (r, i.e., number of shortcuts taken divided by the total number of deadline situations).

### Tests of significance

Again, a Pearson chi-square test [42] was used to test whether the differences between the treatments of varying probabilities of unrealistic deadlines are significant [43]. With the lowest expected frequency count amounting to 68.8 (see Table 4), our data fulfills the criterion of expected frequencies. There is a significant association [Fisher’s exact test; 44] between the probability of software developers being confronted with an unrealistic deadline and the rate of shortcut taking (χ^2^ (3) = 18.788, p < 0.000, Cramer’s V = 0.15). The odds of choosing H were 2.06 times higher in case of p = 0.9 compared to p = 0.4. All odds ratios are shown in Table 5. The general trend is: the higher the probability of being confronted with an unrealistic deadline, the more likely do subjects decide to deliver high quality (in cases they are actually confronted with an unrealistic deadline).

**Table 4:** Contingency table for chi-square test (SPSS output).

			Quality		
			H	L	Total
Treatment	p = 0.4	Count	68	92	160
		Expected count	91.2	68.8	160
		% within treatment	42.5%	57.5%	100%
		% within quality	13.2%	23.7%	17.7%
		% of total	7.5%	10.2%	17.7%
		Std. residual	-2.4	2.8	
	p = 0.5	Count	93	75	168
		Expected count	95.7	72.3	168
		% within treatment	55.4%	44.6%	100%
		% within quality	18.1%	19.3%	18.6%
		% of total	10.3%	8.3%	18.6%
		Std. residual	-0.3	0.3	
	p = 0.7	Count	161	95	256
		Expected count	145.8	110.2	256
		% within treatment	62.9%	37.1%	100%
		% within quality	31.3%	24.4%	28.3%
		% of total	17.8%	10.5%	28.3%
		Std. residual	1.3	-1.4	
	p = 0.9	Count	193	127	320
		Expected count	182.3	137.7	320
		% within treatment	60.3%	39.7%	100%
		% within quality	37.5%	32.6%	35.4%
		% of total	21.3%	14.0%	35.4%
		Std. residual	0.8	-0.9	
Total		Count	515	389	904
		Expected count	515	389	4904
		% within treatment	57.0%	43.0%	100%
		% within quality	100.0%	100.0%	100%
		% of total	57.0%	43.0%	100%

**Table 5:** Odds ratios among the four different treatments.

	p = 0.4	p = 0.5	p = 0.7
p = 0.5	1.68	-	-
p = 0.7	2.29	1.37	-
p = 0.9	2.06	1.23	0.90

To confirm our findings, we further analyzed the data by considering the number of high-quality decisions proportional to all decisions (i.e., we interpreted our categorical variable as the percentage of each participant choosing high quality). However, the Jonckheere-Terpstra test [45, 46] based on the data for each group of two participants showed no significance (p = 0.173).

In this study, we also included a survey as the last part of our experimental design to further connect our experimental results and the game-theoretical predictions by Austin [10]. *Personal traits* from the personal style inventory [48, 49] that have been shown to be relevant in software development before [50] did not lead to any insights. Similarly, our logistic regression analysis [i.e., the suitable test for a categorical dependent variable and categorical as well as continuous independent variables; see 43] did not yield a significant predictor as regards the effect of control variables programming experience, HTML experience, sex, or age.

### Threats to validity

It is worthy of discussion whether a probability of being assigned an unrealistic deadline and the decision of shortcuts are suitable means to represent the relation between time pressure and software quality in its entirety. For instance, quality is also affected by programming errors rather than solely by taking shortcuts that can indirectly lead to errors. However, since we aim to evaluate the game-theoretical prediction concerning time pressure and software quality as proposed by Austin [10], these limitations can be seen as minor threats to *construct validity*.

Having professional software developers participate in the experiment instead of students would have increased the *external validity*, but professional developers’ time restrictions and payment requirements make it unlikely to find a sufficient number of participants. The framing design of the experiment ensures that external validity is not threatened by differences in specific programming skills between students and professionals (see Section “Experimental Studies, General Setup” for a detailed discussion.)

*Internal validity* is threatened due to the framing design of our experiment. While subjects in the first study were not confronted with a real programming task, the programming task in the second study were minor ones and limited to a markup language. However, the actual decision of software developers as well as our experimental subjects (whether to report a delay or take a shortcut in case of an unrealistic deadline) is made based on the given conditions (p, Q_1_, Q_2_, C, and the assigned deadline situation). Therefore, this decision mostly takes place *before* the actual implementation of the assigned task is carried out. We are thus confident to have constructed a sufficient representation of a real-live setting. Furthermore, we believe that framing was the only viable option in our setting. Our first study uses the clearest representation of the developers’ strategic situation. Our second study gives up some experimental control over the agents’ costs (e.g., for the effort of conducting the task vs. taking a shortcut) in order to introduce a real programming task and thus to gain internal validity.

## Discussion

We contribute to the understanding of the relationship between time pressure and performance in work context in order to help resolve the theoretical and empirical inconsistencies in this regard. Our study corroborates the game-theoretical prediction by Austin [10] to the extent that for specific high probabilities of unrealistic deadlines software developers decide to deliver high quality. Further, the relation between the probability of unrealistic deadlines and the share of high-quality decisions (based on the chi-square test) is in favor of a monotone relation. Finally, our study sheds light on the empirical value of p_crit_ to avoid shortcut taking in software projects.

### Theoretical implications

Our study continues research on the impact of time pressure on performance since it was motivated by the inconclusiveness concerning the dyadic relationship between time pressure and performance in general and set out to find evidence to support or refute the game-theoretical prediction by Austin [10] concerning the impact of deadlines on software quality in particular. Austin’s model comes along with the hypothesis that the rate of shortcuts taken in software development projects can be reduced by setting deadlines aggressively (i.e., the rate of unrealistic deadlines p should be larger than a critical value p_crit_). The experiment parameters were set to imply a p_crit_ of 0.5. In our analysis, we found that taking shortcuts is the prevailing decision for p = 0.4 in both experiments. In contrast, shortcuts were almost completely eliminated for p = 0.9 in the first experiment (see Figs 3 and 5) and were shown to decrease in the second experiment (i.e., the rates of shortcut taking for p > p_crit_ are lower than for p < = p_crit_; see Fig 9). For p between 0.5 and 1, Austin also stipulates a shortcut rate of zero. In the experiments, however, we found no clear pattern. There were subjects that were mostly indecisive whether to take shortcuts. Our results are thereby in line with studies suggesting that different levels of time pressure have different kinds of impact, that is, a non-linear relationship between time pressure and performance [6]. According to Austin [10], there should be a linear increase of shortcuts taken for probabilities lower than p_crit_ and no shortcut taking for probabilities equal to or greater than p_crit_. In general, we find a trend that shows a decreasing shortcut-taking rate along the increase of p (i.e., the rates of shortcut taking for p > p_crit_ are below the rates of shortcut taking for p < = p_crit_). Shortcut taking frequently takes place for p = 0.4 and can be significantly decreased for p = 0.9. The general trend is that higher probabilities of unrealistic deadlines are linked to higher software quality.

### Limitations and directions for future research

We see several design decisions that are worth a discussion concerning our experimental setting. First, the game-theoretical model and our experimental setting presume p to be equal and independent for both developers. Thus, the skills of the developers are likely to be decisive for whether a deadline can be met. Whereas our experiment is designed as a framing one, a real-life evaluation in the field within a software development company might lead to different insights. Software developers might use shortcuts in cases of realistic deadlines as well or increase capacity (e.g., night shifts) to deliver high quality when facing unrealistic deadlines.

Software developers might be involved in effort estimation [51] and thus have a notion of the actual deadlines. This knowledge is likely to mitigate the effect of time pressure. Moreover, close collaboration and intensive communication among team members [52, 53] is relevant, particularly in case of agile development approaches [54–56], which are designed for intensive interaction, thus enabling an extensive social exchange. Accordingly, we suggest that the scenario described by Austin [10] is less likely to apply to such more recent development approaches. Austin’s theoretical results and our empirical evaluation are more likely to apply to more traditional approaches of software development. That said, the model and our findings are not irrelevant for industrial practice–many companies mix and match development approaches and tailor methods to fit their particular needs [57]. Thus, for example, you see stage-gate models (i.e., using phased delivery deadlines for releases) blended with agile approaches in industrial practice [58].

The findings show that continuously having aggressive deadlines in software development projects might indeed be effective for improving the quality of the products. Whereas reporting delays in software projects becomes destigmatized, shortcut taking only completely vanishes at p = 0.9 in our first study. Such a high rate of unrealistic deadlines might be unworkable. For lower values of p_crit_ to be effective, quality and career penalties could be altered. This could be done for example by communicating a corporate identity to the software developers which particularly values the quality of the software products [15] and makes employees profit from high quality rather than from meeting deadlines.

Previous research has shown that time pressure decreases decision quality [3, 13, 59], thus contradicting Austin’s analytical results. However, studies likewise show that decision quality increases if pressure is generated by work-pace dependent incentives (i.e., higher payoff if faster) [3]. In this context, it is important to consider how time pressure is created. Our experiment was designed in a way that software developers face an unrealistic deadline. The approach of allocating insufficient time [27] in general is problematic because different individuals work at a different pace. Since we used a framing for our experiment, this problem did not arise in our setting.

While we were not able to determine the concrete value of p_crit_ due to our study design, we falsified the theoretical value for p_crit_, which according to Austin’s model and our parameter choices should have been 0.5. We call upon further research to determine the actual critical value of p and analyze reasons for the deviation between theory and empirical evidence. In addition, researchers should address settings with more than two software developers because team structure can have an impact on perceived time pressure [15]. Additionally, future research should consider additional options for designing the experiment such as varying incentive mechanisms and different punishment levels for not meeting the deadline.

### Managerial implications

Our study provides implications that are relevant to the management of software projects. First, traditional ways of deadline setting should be reconsidered. It is likely that shortcuts taken by software developers can be reduced if unrealistic deadlines are seen as an ever-present condition in the organization. While other recommendations suggest that time pressure should not exceed a specific level to avoid negative consequences like distraction, our results are in favor of a constantly high level of time pressure. However, our findings should not be seen as normative. We have analyzed how given levels of time pressure affect shortcut taking in software development projects. The actual scarcity level of the available time in relation to the time required to accomplish a task is subject to further investigations.

Second, our study suggests that the value of p_crit_ that leads to the desired effect of software developers choosing high quality over shortcuts is rather high. Consequently, the consonance of such a high level of time pressure with organizations’ ethical principles and work culture needs to be examined. Once developers become aware that deadlines are most likely unrealistic, the role of time pressure as a motivator [e.g., 24] might vanish. Thus, the actual level of time pressure applied in organizations needs careful consideration in the given context. In this regard, the experience of the developers needs to be considered as well.

Finally, organizations need to carefully consider whether they follow the deadline-setting policy advocated by the game-theoretical analysis of Austin (2001) or choose to seek a more transparent approach, particularly in agile development environments. Software development is a social activity and managers are unlikely to prevent their developers from discussing deadlines and the respective policies. In particular, agile development approaches benefit from the interaction of all project members (e.g., in daily scrums).

## Conclusion

This study helps to dissolve existing inconsistencies concerning the relationship between time pressure and work performance by reporting the design and results of an experiment to test the widely disseminated and discussed hypothesis by Austin [10] that increasing time pressure leads to improved quality in software projects. Within this study, we indicate that a critical probability exists for assigning unrealistic deadlines to software developers. Exceeding this critical probability leads to a considerable reduction of shortcut taking and thus higher software quality. Whereas this finding corroborates the game-theoretical hypothesis derived by Austin [10], we also show that the actual critical probability is above the theoretical one. Accordingly, shortcuts are likely to be avoided only if software developers perceive unrealistic deadlines as an ever-present condition in the organization. In this regard, the programming experience can be seen as a factor that supports taking shortcuts and thus leads to a higher empirical value of p_crit_. While a final conclusion on the impact of time pressure on software quality requires further research varying the context and the way time pressure is created, our results suggest that time pressure can be software engineering’s best friend rather than the greatest enemy in the attempt to ensure quality in software projects.

## Supporting information

S1 Appendix Analysis of Austin’s agency model. (DOCX)

S2 Appendix Study 1: Setting, instructions, procedure, and control questions. (DOCX)

S3 Appendix Study 2: Setting, instructions, procedure, control questions, HTML tutorial, and experimental tasks. (DOCX)
