# Supplementary material for: Impact of time pressure on software quality: A laboratory experiment on a game-theoretical model
Source: PLoS One. 2021 Jan 15;16(1):e0245599. doi: 10.1371/journal.pone.0245599 (PMC7810279; doi:10.1371/journal.pone.0245599)
Supplement: S1 Appendix — (DOCX) [file pone.0245599.s001.docx]

## Analysis of Austin’s agency model.

Presume p ∊ (0,1] to exclude the trivial game that always ends with penalties (0, 0).

We derive the normal form (NF) of the game, as given in Table 1, by calculating the players’ expected payoffs, that is, expected penalties, u_1_ and u_2_ from each strategy profile. For example, for the strategy profile (H, L), the expected penalties of agent 1 are u_1_(H, L) = p^2^(C+Q_1_)+p(1-p)C+(1-p)pQ_1_+(1-p)^2^0=p(C+Q_1_).

| Table 1. NF of Austin’s Game | | | |
| --- | --- | --- | --- |
|  | H | L | |
| H | p(1-p)C | pQ_1_ | |
|  | p(1-p)C | p(C+Q_1_) | |
| L | p(C+Q_1_) | p^2^Q_2_+2p(1-p)Q_1_ | |
|  | pQ_1_ | p^2^Q_2_+2p(1-p)Q_1_ | |
| Agent 1: Row player  Agent 2: Column player | | Payoffs in cells: | u_2_  u_1_ |

In contrast to the NF in Table 1, the NF presented by Austin (2001) contains for each agent her expected penalties from a strategy profile conditional on her facing an unrealistic deadline and ignores the negative expected externality (1-p)pQ_1_ that arises if the opponent chooses L. In personal correspondence, Robert Austin explained that his original manuscript contained a footnote explaining the reason for choosing his NF. That footnote, however, was removed for reasons of length and the insight that the difference was of no consequence in terms of his strategic analysis (cf., e.g., Mas-Colell et al., 1995 for a proof of equivalence of the two approaches – using the conditional or the unconditional payoffs – for the Bayes Nash equilibrium analysis). For our analysis, we prefer to stick with the usual NF in Table 1 (cf. Mas-Colell et al., 1995), which contains the ex-ante (unconditional) expected payoffs from each strategy profile. The penalty comparisons that are relevant for deriving pure-strategy Bayesian Nash equilibria are unaffected (and therefore p_crit_ is the same). However, payoff comparisons in the analysis of Pareto optimality and payoff dominance are affected. For instance, to tell which equilibrium payoffs are Pareto-optimal, we need to compare both players’ expected payoffs from the respective equilibrium strategy profile, that is, the ex-ante expected payoffs (incl. externalities resulting from the decision of the other player) – rather than only those that apply after being informed of an unrealistic deadline, that is, rather than both players’ conditional payoffs that potentially apply different conditions. The negative externality (1-p)pQ_1_ leads to the multipliers “2” in the formula for p_ps_ (see below), which are not present in Austin’s threshold on Pareto-superiority (but p_crit_ > p_ps_ holds also in Austin’s analysis). Nevertheless, we emphasize that the equilibria and the fundamental insights from the game and the experiment are not affected by the difference between the analyses.

From the NF, we derive all pure-strategy Bayesian-Nash equilibria (NEQ):

*(L, L) is a NEQ for all p.*

Proof. Unilaterally deviating from (L, L) is not profitable, thus (L, L) is a NEQ. The penalties are given in Table 1. The expected penalties from playing L if the opponent plays L are lower than those from playing H against L for player i ∊ {1,2}:

$p^{2}Q_{2}+2p\left( 1-p \right)Q_{1}=\left( \begin{matrix} p^{2} \\ p\left( 1-p \right) \\ \left( 1-p \right)p \\ \left( 1-p \right)^{2} \end{matrix} \right)\left( \begin{matrix} Q_{2} \\ Q_{1} \\ Q_{1} \\ 0 \end{matrix} \right)\leq\left( \begin{matrix} p^{2} \\ p\left( 1-p \right) \\ \left( 1-p \right)p \\ \left( 1-p \right)^{2} \end{matrix} \right)\left( \begin{matrix} C+Q_{1} \\ C \\ Q_{1} \\ 0 \end{matrix} \right)=p(C+Q_{1})$.

This condition is true for all p, which can be deduced by comparing the cost terms pairwise: Q_2_ < C + Q_1_, Q_1_ < C. For all p ∊ (0,1], the inequality holds strictly, that is, L is the unique best response to L. ■

*(H, H) is a NEQ iff p ≥ (C-Q_1_)/C =: p_crit_. Note, 1 > p_crit_ > 0.*

Proof. Unilaterally deviating from (H, H) is not profitable, thus (H, H) is a NEQ. The expected penalties for player i ∊ {1,2} from playing H if the opponent plays H are lower than those from playing L against H (cf. Table 1):

$$p\left( 1-p \right)C\leq pQ_{1}.$$

For p > 0, rearranging terms gives p ≥ (C-Q_1_)/C = p_crit_. By C > Q_1_ > 0, 1 > p_crit_ > 0. ■

*These are all NEQ, so for all p, (H, L) and (L, H) are not NEQ.*

Proof. As shown in the proof of (L, L) being a NEQ, L is the unique best response to L for all p ∊ (0,1]. ■

A popular criterion to select among multiple NEQ is payoff dominance (Harsanyi & Selten, 1988).

*If both (L, L) and (H, H) are NEQ, (H, H) is payoff-dominant to (L, L).*

Proof. We know that both (L, L) and (H, H) are NEQ iff p ≥ p_crit_ = $\frac{C-Q_{1}}{C}$. The NEQ (H, H) payoff-dominates the NEQ (L, L) if, for both players, the penalty from the NEQ (H, H) is lower than the penalty from the NEQ (L, L) (cf. Table 1):

$p\left( 1-p \right)C< p^{2}Q_{2}+2p\left( 1-p \right)Q_{1}$.

Rewriting, we retrieve

$p>\max\left\{ \frac{C-2Q_{1}}{C-2Q_{1}+Q_{2}},0 \right\}=:p_{ps}$.

This inequality holds whenever both (L, L) and (H, H) are NEQ because p_crit_  > p_ps_:

$\frac{C-Q_{1}}{C}>\max\left\{ \frac{C-2Q_{1}}{C-2Q_{1}+Q_{2}},0 \right\}\boldsymbol{\Leftarrow}\left( Q_{2}-Q_{1} \right)C+Q_{1}^{2}>\left( Q_{2}-Q_{1} \right)Q_{1}$ and $\frac{C-Q_{1}}{C}>0,$

which are both true because C > Q_2_ > Q_1_ > 0. ■

Furthermore, the payoffs from (H, H) are the unique Pareto-optimal payoffs from the game iff p ≥ p_crit_ (i.e., iff (H, H) is a NEQ). Also note that L is a strictly dominant strategy iff p < p_crit_.

Based on these strategic properties, three well-known games can be distinguished, depending on p and the penalties:

- p ∈ (0, p_ps_]: A game with exactly one NEQ (L, L) and Pareto-optimal equilibrium payoffs. In this game, L is a strictly dominant strategy such that rational players will always prefer L. This case only exists for C > 2Q_1_ because p_ps_ > 0 iff C > 2Q_1_. (The denominator in pps = max{(C-2Q1)/(C-2Q1+Q2),0} is always > 0 because C > Q1 and Q2 > Q1.)
- p ∈ (p_ps_, p_crit_): A Prisoner’s Dilemma with the NEQ (L, L) and Pareto-dominated equilibrium payoffs. This is the classic situation in which L is strictly dominant such that players have individual incentives to play L, yet both would benefit if coordinating to play H.
- p ∈ [p_crit_, 1]: A Stag Hunt, which is, a coordination game with two NEQs, here (H, H) and (L, L), with the first payoff-dominating the second.

The above analysis presumes risk-neutral agents. However, the agents’ risk attitude impacts p_crit_, which is the threshold on p such that for lower p an agent prefers a penalty Q_1_ over a lottery that yields a penalty C with probability (1-p) and no penalty with probability p. A risk-neutral agent therefore prefers the penalty over the lottery for all p ≤ (C-Q_1_)/C. Agents with an aversion to risk prefer the certain penalty to the lottery for a larger range of p. With such agents, p_crit_ must therefore be higher (and the inverse holds for risk-seeking agents).

Agents may play Austin’s game not only once but also repeatedly. If a pair of agents plays the same game repeatedly, the history-independent repeated play of the NEQ strategies of the one-shot game forms a (subgame-perfect) NEQ of this enhanced game (Fudenberg & Tirole, 1991). Thus, in the repeated version of Austin’s game, play of L by each agent in each round is a NEQ for p < p_crit_, and play of H in each round is the payoff-dominant NEQ for p ≥ p_crit_, and agents choose these actions independent of what they observed in previous rounds.
