# Supplementary material for: Impact of time pressure on software quality: A laboratory experiment on a game-theoretical model
Source: PLoS One. 2021 Jan 15;16(1):e0245599. doi: 10.1371/journal.pone.0245599 (PMC7810279; doi:10.1371/journal.pone.0245599)
Supplement: S2 Appendix — (DOCX) [file pone.0245599.s002.docx]

# Study 1: Setting, instructions, procedure, and control questions^[[1]](#footnote-1)^

We kindly welcome you to this economic experiment. It is very important that you read the following explanations carefully. If you have any questions, please raise your hand and we will come to your place and answer your questions.

Setting

In this experiment, you can earn money depending on your decisions and the decisions of the other participants. During the experiment, you are not allowed to communicate with any of the participants. Failure to comply with this rule leads to exclusion from the experiment and from all payments. All decisions are made anonymously, that is, none of the other participants will know the identity of the person who made a particular decision. The payout is also anonymous, that is, no participant will know how high the payout of the other participants is.

During the experiment, your total income is calculated in points. The total number of points you earn during the experiment will then be converted into EUR at the end of the experiment, whereby 20 points correspond to 1 EUR (rounded up to the next 10 cents). At the end of today's experiment, we will pay you the number of points earned during the experiment (converted into EUR) plus 2.50 EUR for your appearance in cash. On the following pages, we explain the exact procedure of the experiment.

Instructions

Imagine being a software developer in a company. There is one manager and another software developer (hereinafter referred to as your colleague). In the course of time, you and your colleague receive tasks from the manager at the same time and repeatedly and compete with your colleague for the favor of the manager (in the form of bonus payments, career opportunities, etc.).

The manager sets a deadline for each subtask, which can be either realistic or unrealistic. Experience has shown that the probability of getting an unrealistic deadline is 40% / 60% / 75% / 90%^[[2]](#footnote-2)^. This applies both to you and your colleague. As soon as you receive a concrete task, you know whether this task has a realistic or an unrealistic deadline. However, you do not know whether your colleague's deadline is realistic or unrealistic, since it is independent of yours (but the same probability of an unrealistic deadline of 40% / 60% / 75% / 90% applies). If you have received a realistic deadline, you deliver the programming task on time as required (i.e., in high quality). In case of an unrealistic deadline, you have to make a decision:

Option 1: You can still complete the programming task on time by deliberately delivering the software in low quality. This means that you do not deliver the task with your best conscience, but with shortcomings that can lead to an error in the operation of the software. An example of such an inadequacy is an incomplete exception-handling mechanism. Assume that the manager cannot recognize the low quality as such because she does not have the necessary expertise to review the software code. Also assume that a potential error cannot be traced back to you specifically. However, the mistake would indirectly harm both you and your colleague, as it would damage the image of the company for which you both work.

Option 2: You can deliver the programming task in high quality but late. This means that you must notify the manager of the delay. The consequence can be a career disadvantage for you, namely when your colleague delivers the task on time, because in this case you perform poorly in the eyes of the manager compared to your colleague. You consider the potential personal disadvantage to be more serious than the indirect damage in case of low quality. On the other hand, this also means that you and your colleague can avoid any penalties (career and quality) if both get an unrealistic deadline and still deliver high quality.

Procedure

- The experiment consists of 2 * 24 = 48 rounds, with each round having the same structure. One round corresponds to a software project, that is, one deadline situation.
- For the first 24 rounds you will be randomly assigned to a colleague. After 24 rounds you will be randomly assigned to another colleague.
- In the event of a realistic deadline, you have no decision to make. You deliver the programming task with high quality on time.
- In the case of an unrealistic deadline, you can now choose between “High quality (i.e., delay)” and “Low quality (i.e., no delay)”.
- In each round (i.e., for each software project), you will first receive a basic reward of 8 points, regardless of the deadline situations and decisions. Deadline situations and decisions have the following monetary implications:
  - If you choose high quality with an unrealistic deadline, that is, you report the delay to the manager and process the task, but your colleague does not, you will suffer a personal career disadvantage (penalty) of 4 points. Conversely, this also applies to your colleagues. If both deliver high quality with unrealistic deadlines at the same time (by sending a corresponding message to the manager and processing the task), there is no penalty.
  - If exactly one of you delivers low quality, this harms the image of the company, which is reflected in a penalty of 2 points for you and your colleague. If both deliver low quality, this leads to a penalty of 3 points each.
- Please be aware:
  - You can lose points even though you get a realistic deadline and cannot actively make a decision, namely when your colleague delivers low quality.
  - There are two possible cases when your colleague delivers high quality: (1) She has a realistic deadline or (2) she has an unrealistic deadline and opts for high quality (report delay).
  - There are two possible cases when you suffer a career disadvantage by reporting a delay to the manager: Your colleague has opted for low quality with a similarly unrealistic deadline or your colleague has had a realistic deadline (and can therefore deliver high quality).
- Consider your decision carefully before you make it on the screen.
- At the end of each round (after your decision), you will receive the following information on the screen:
  - Your score before the decision
  - Your decision
  - Whether your colleague has delivered high or low quality
  - Your current score
- The following decision tree summarizes the possible situations and associated penalties for you and your colleague:

Control questions

1. How many rounds are played in total?
2. How many rounds does your colleague stay the same?
3. With how many colleagues do you interact during the experiment?
4. What is the probability of an unrealistic deadline for you?
5. What is the probability of an unrealistic deadline for your colleague?
6. Does your colleague's deadline situation depend on your own deadline situation?
7. Do you know your own deadline situation?
8. Do you know the deadline situation of your colleague?
9. Do you have to make a decision in each round?
10. Please enter your penalties in the following table in the respective situations. Please also enter the indicated probabilities of occurrence.

1. The study was conducted using the z-Tree software package (Zurich Toolbox for Readymade Economic Experiments; cf. Fischbacher, 2007). [↑](#footnote-ref-1)
2. Only one of those values was given to the participants (depending on the treatment). Same applies further below. [↑](#footnote-ref-2)
