# Supplementary material for: Impact of time pressure on software quality: A laboratory experiment on a game-theoretical model
Source: PLoS One. 2021 Jan 15;16(1):e0245599. doi: 10.1371/journal.pone.0245599 (PMC7810279; doi:10.1371/journal.pone.0245599)
Supplement: S3 Appendix — (DOCX) [file pone.0245599.s003.docx]

# Study 2: Setting, instructions, procedure, control questions, html tutorial, and experimental tasks^[[1]](#footnote-1)^

Dear participant, we kindly welcome you to this economic experiment. It is very important that you read the following explanations carefully. If you have any questions at the end of this introduction, please raise your hand and we will come to your place and answer your questions.

Setting

In this experiment, you can earn money depending on your decisions and the decisions of the other participants. During the experiment, you are not allowed to communicate with any of the participants. Failure to comply with this rule leads to exclusion from the experiment and from all payments. All decisions are made anonymously, that is, none of the other participants will know the identity of the person who made a particular decision. The payout is also anonymous, that is, no participant will know how high the payout of the other participants is.

During the experiment, your total income is first calculated in points and then converted into EUR. The conversion factor for points into EUR is 6:1 and you will also receive a show-up fee of 4.00 EUR.

Instructions

Imagine being a software developer in a company. There is one manager and another software developer (hereinafter referred to as your colleague). In the course of time, you and your colleague receive tasks from the manager at the same time and repeatedly and compete with your colleague for the favor of the manager (in the form of bonus payments, career opportunities, etc.). Together with your colleague you work on the development of an online shop and create a website for it. The manager sets a deadline for each subtask, which can be either realistic or unrealistic. Experience has shown that the probability of getting an unrealistic deadline is 40% / 50% / 70% / 90%.^[[2]](#footnote-2)^ This applies both to you and your colleague. As soon as you receive a concrete task, you know whether this task has a realistic or an unrealistic deadline. However, you do not know whether your colleague's deadline is realistic or unrealistic, since it is independent of yours (but the same probability of an unrealistic deadline of 40% / 60% / 75% / 90% applies). If you have received a realistic deadline, you can begin processing the task afterwards. In case of an unrealistic deadline, you have to make a decision:

Option 1: You can still complete the programming task on time by deliberately delivering the website in low quality. In concrete terms, this means that you copy an existing solution from another online shop, but with minor bugs.^[[3]](#footnote-3)^ This means that you do not deliver the task with your best conscience, but with shortcomings that can lead to an error in the operation of the website. Assume that the manager cannot recognize the low quality as such because she does not have the necessary expertise to review the HTML code. Also assume that a potential error cannot be traced back to you specifically. However, the mistake would indirectly harm both you and your colleague, as it would damage the image of the company for which you both work.

Option 2: You can deliver the programming task in high quality but late. This means that you must notify the manager of the delay. The consequence can be a career disadvantage for you, namely when your colleague delivers the task on time, because in this case you perform poorly in the eyes of the manager compared to your colleague. You consider the potential personal disadvantage to be more serious than the indirect damage in case of low quality. On the other hand, this also means that you and your colleague can avoid any penalties (career and quality) if both get an unrealistic deadline and still deliver high quality.

If both have a realistic deadline, both deliver high quality.

Procedure

- The experiment consists of 10-15 rounds, with each round having the same structure. The exact number of rounds will only be announced after the last round has been completed. Before the first round, you will be randomly assigned to a colleague. This colleague remains the same for all rounds. A round corresponds to one subtask, that is, one deadline situation.
- In the event of a realistic deadline, you have no decision to make. You can work on the task without time pressure.
- In the event of an unrealistic deadline, it is impossible for you to complete the task in the given time. Instead, you now have to choose between two options: "Edit" (i.e., report delay and deliver high quality without time pressure) and "Copy" (i.e., not report a delay but copy a poor-quality solution).
- In each round (i.e., for each subtask), you will first receive a basic reward of 8 points, regardless of the deadline situations and decisions. The number of points you will receive in each round depends on how many points are deducted from the 8 starting points. The penalty depends on the deadline situations and your decisions as follows:
  - If you choose high quality with an unrealistic deadline, that is, you report the delay to the manager and process the task, but your colleague does not, you will suffer a personal career disadvantage (penalty) of 4 points. Conversely, this also applies to your colleagues. If both deliver high quality with unrealistic deadlines at the same time (by sending a corresponding message to the manager and processing the task), there is no penalty.
  - If exactly one of you delivers low quality, this harms the image of the company, which is reflected in a penalty of 2 points for you and your colleague. If both deliver low quality, this leads to a penalty of 3 points each.
- Please be aware:
  - You can lose points even though you get a realistic deadline and cannot actively make a decision, namely when your colleague delivers low quality.
  - There are two possible cases when your colleague delivers high quality: (1) He/she has a realistic deadline or (2) he/she has an unrealistic deadline and opts for high quality (report delay).
  - There are two possible cases when you suffer a career disadvantage by reporting a delay to the manager: Your colleague has opted for low quality with a similarly unrealistic deadline or your colleague has had a realistic deadline (and can therefore deliver high quality).
- Consider your decision carefully before you make it on the screen.
- At the end of each round (after your decision and the eventual processing of the task), you will receive the following information on the screen:
  - Your decision
  - Your colleague's action (but not his or her underlying deadline situation)
  - Your points from this round
  - Your current total score
- The following decision tree summarizes the possible situations and associated penalties for you and your colleague:

Control questions

1. How many rounds are played in total?
2. How many rounds does your colleague stay the same?
3. With how many colleagues do you interact during the experiment?
4. What is the probability of an unrealistic deadline for you?
5. What is the probability of an unrealistic deadline for your colleague?
6. Does your colleague's deadline situation depend on your own deadline situation?
7. Do you know your own deadline situation?
8. Do you know the deadline situation of your colleague?
9. Do you have to make a decision in each round?
10. How do you proceed if, in case of an unrealistic deadline, you still want to complete a task in the given time?
11. Can you deduce your colleague's underlying deadline situation from her/his action (editing vs. copying)?
12. Please enter your penalties in the following table in the respective situations. Please also enter the indicated probabilities of occurrence.

HTML Tutorial

1. Basics and Structure

HTML (**H**yper**t**ext **M**arkup **L**anguage) is the language in which web pages are written. This language describes how a page looks in the form of so-called tags. The following example shows an opening tag <x> and a closing tag </x> that enclose a word.

<x>example</x>

The browser interprets the tags and presents the included text accordingly. A tag can be used, for example, to include an image or to bold the text in a certain area.

Basically, an HTML page is structured as follows:

<html>

<head>

</head>

<body>

</body>

</html>

The <html> tags mark the beginning and end of the HTML document. There are also two important sections with defined contents: the head and body of the document.

The header of the HTML page contains the meta information about the page, for example, the title of the page that is shown in the browser window header. Specify a title by entering <title> in the tag:

<title>Your page title</title>

The actual content of the page, which will be displayed later, is in the body.

1. Text Formatting

Within the body, you create a simple text segment (e.g., a word or sentence) with the tag <p>:

<p>example text</p>

You can format the text itself in **bold** (with <b>), *italic* (with <i>), or underlined (with <u>):

<b>fat</b>.

<i>italic</i>

<u>underlined</u>

You can also use predefined tags for headlines of different sizes:

<h1>heading 1</h1>

<h2>heading 2</h2>

<h3>heading 3</h3>

1. Elements

3.1 Tables

In HTML, a table consists of table rows (<tr>), which contain table cells (<td>). In addition, special table cells can be defined for the header line (<th>). The **border** attribute within the <table> tag can be used to define a frame.

Example:

| **Category 1** | **Category 2** |
| --- | --- |
| Cell 1 | Cell 2 |

<table border="1">

<tr>

<th>Category 1</th>

<th>Category 2</th>

</tr>

<tr>

<td>Cell 1</td>

<td>Cell 2</td>

</tr>

</table>

3.2 Graphics

Add pictures via the tag <img>. You can specify additional attributes within this tag: **src** specifies the path under which the image file can be found, **alt** defines an alternative text if the image cannot be displayed. Attention: There is no closing tag for graphics.

<img src="URL.png" alt="Alternate text">

3.3 Buttons

You can create a button that the user can press by using the <button> tag as follows:

<button>example-button</button>

This button is interpreted differently in different browsers. To prevent errors, it is therefore possible to declare the type of the button with the attribute **type** as a "button" within the tag:

<button type="button">example button</button>

Experimental Tasks

Below, we show the tasks the participants were asked to accomplish during the experiment (one task per round). In case of a realistic deadline, the instructions read as follows:

“Your manager has given you sufficient time to complete the task. Therefore, the deadline is realistic and you can edit the task.”

In case of an unrealistic deadline, the instructions read as follows:

“Your manager has given you 10 seconds to complete the task. This deadline is unrealistic and cannot be met by you under any circumstances. You can

- ask your boss for an extended deadline and complete the task. In this case, you have enough time for the processing and can deliver high quality.
- Alternatively, you can copy a solution that a colleague worked out a few weeks ago. This solution contains minor bugs, so you deliver lower quality.”

1. Build the HTML structure consisting of head and body (including the outer <html> tags).
2. Please insert the following title for your website: My Online Shop
3. Please insert the following heading <h1>: Range of Products
4. Insert the following text section: Welcome to our online shop! Put the items you want to buy in your shopping cart.
5. Create a table and define a frame of size 1.
6. Add column headers (<th>) to the table. Create four columns: ID, name, picture, order.
7. Create a table row <tr>. Leave the fourth cell (order) blank, but create it anyway.
   Column 'ID': 1
   Column 'name': Table
   Column 'image': <img src="table. jpg">
   Column 'order': empty
8. Under the column heading "order", create a simple button in the created row, which can be used to order the product. Label this button with the following title: Add to shopping cart
9. Add the following alternative text for the inserted image: Image of a table
   To do this, adjust the following line of code:
   <img src="Table.jpg">
10. In order to function consistently across all browsers, the button type (type ="button") must be defined. To do this, adjust the following line of code:
    <button>Add to shopping cart</button>
11. Insert the following text underneath the table: Shipping within Germany is 4.50 EUR.
    The word ‘shipping’ should be bold formatted and ‘4.50 EUR should be underlined.

1. The study was conducted using the oTree software (Chen, Schonger, & Wickens, 2016), which is implemented in HTML and thus better suited for our setting compared to zTree (used in *Study 1*). [↑](#footnote-ref-1)
2. Only one of those values was given to the participants (depending on the treatment). Same applies further below. [↑](#footnote-ref-2)
3. Additional note: The participants were offered a button that allowed them to directly copy the source code from a different project to theirs. [↑](#footnote-ref-3)
